# Methicillin-Resistant Staphylococcus aureus Renal Abscess Unmasks Human Immunodeficiency Virus Infection in an Adolescent: A Case Report

**DOI:** 10.7759/cureus.87112

**Published:** 2025-07-01

**Authors:** Lubana Thasneem, Manasa Reddy, Mounika Reddy, Abhishek J Arora, Madhusudan Samprathi

**Affiliations:** 1 Pediatrics, All India Institute of Medical Sciences, Bibinagar, Hyderabad, IND; 2 Pediatric Surgery, All India Institute of Medical Sciences, Bibinagar, Hyderabad, IND; 3 Radiodiagnosis, All India Institute of Medical Sciences, Bibinagar, Hyderabad, IND

**Keywords:** community-acquired, highly active antiretroviral therapy, immunodeficiency, pediatric, percutaneous drainage, vancomycin

## Abstract

Renal abscesses are rare in children. This report presents a case where a renal abscess due to methicillin-resistant *Staphylococcus aureus* (MRSA) was the initial indication of immunocompromised status in an adolescent boy. A 17-year-old male patient presented with a month-long history of fever, fatigue, and significant weight loss, along with dull aches and swelling in the left flank. There was no history of urinary symptoms, vomiting, or high-risk behaviors. His mother had died from sepsis while on antiretroviral therapy (ART), and his father from coronary artery disease. The patient had not been previously tested for human immunodeficiency virus (HIV). Examination revealed a tender fullness in the left lumbar region. Ultrasound showed a collection in the left kidney. Blood tests indicated anemia and elevated inflammatory markers. The patient tested positive for HIV antibodies. Empirical antibiotics were started. Ultrasound-guided aspiration grew MRSA, prompting a switch to vancomycin. A percutaneous catheter was inserted due to abscess recurrence. The patient received intravenous vancomycin followed by oral linezolid and started antiretroviral therapy. Repeat imaging at six weeks showed resolution of the abscess. Renal abscesses in children can be life-threatening and often result from urinary tract infections or hematogenous spread. This patient's abscess likely resulted from hematogenous spread due to immunocompromised status. Effective management, including prompt percutaneous drainage and targeted antibiotics, combined with ART, led to abscess resolution and a favorable outcome. This case underscores the importance of considering immunodeficiency in pediatric patients with unusual infections like renal abscesses and highlights the necessity of a multidisciplinary approach and aggressive management for complex infections in immunocompromised children.

## Introduction

Renal abscess is a rare but serious condition in children, accounting for less than 1% of all pediatric renal infections and associated with significant morbidity and mortality if not promptly diagnosed and treated [1,2]. Clinical presentation is often nonspecific, making early recognition challenging. *Staphylococcus aureus*, including methicillin-resistant strains, is a causative pathogen, particularly in the context of skin and soft tissue infections, indwelling devices, or immunocompromised states [3]. In some cases, severe or atypical infections may be the first clinical sign of an underlying immunodeficiency [4]. Renal abscesses have been rarely reported as the initial presentation of human immunodeficiency virus (HIV) infection in children and adolescents.

Here, we present a rare case where renal abscess, attributed to methicillin-resistant *S. aureus* (MRSA), served as the initial indication of immunocompromised status in an adolescent boy. The patient responded favourably to treatment, which included percutaneous drainage and intravenous antibiotics. This emphasizes the need to consider immunocompromisation in pediatric patients presenting with renal abscess, especially in the absence of known urological anomalies or recent procedures. Timely imaging and intervention are essential for effective management and prevention of long-term renal complications. This case illustrates the complexity and challenges of managing renal abscess in a pediatric patient with an underlying immunocompromised status.

## Case presentation

A 17-year-old male patient was hospitalized with a month-long history of recurring fever, especially in the evenings, accompanied by fatigue and a significant weight loss of 6-7 kg. He also reported increasing, persistent dull aches in the left flank along with swelling for about 15 days. There was no history of hematuria, dysuria, pyuria, frequency or urgency of micturition, vomiting, diarrhea, jaundice, joint pains, persistent cough, rapid breathing, or loss of appetite. There was no history of recurrent infections in the past, hospitalizations, surgeries, or blood transfusions. The patient denied recent antibiotic use, urinary instrumentation, trauma, or engagement in high-risk behaviors such as sexual activity or intravenous drug use. In the patient’s family medical history, his mother passed away a year and a half ago due to a leg infection progressing to sepsis while on antiretroviral therapy (ART). His father, who tested negative for HIV infection, died two years ago due to coronary artery disease. The patient had not been previously tested for HIV infection.

**Figure 1:**
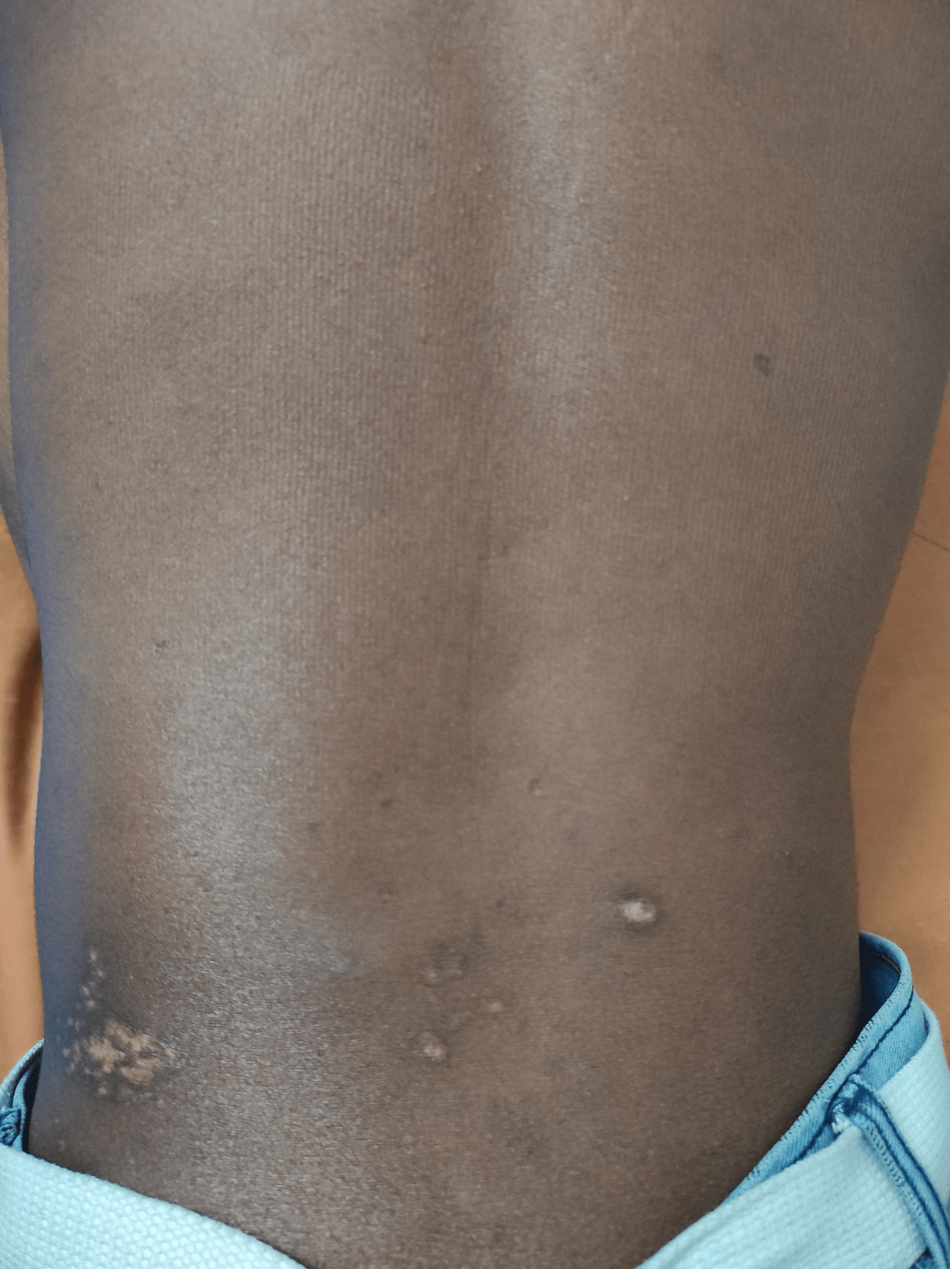
Clinical image showing diffuse fullness in the left lumbar region with the overlying skin showing areas of altered pigmentation.

On examination, he had a temperature of 97.30°F, a heart rate of 102 beats per minute, a respiratory rate of 20 breaths per minute, and a blood pressure of 100/68 mmHg. Despite the weight loss, he appeared well and in good health, weighing 46 kg (10th centile), with a height of 160 cm (between 3rd and 10th centile). He had pallor; there was no icterus, clubbing, cyanosis, pedal edema, lymphadenopathy, or oral thrush. The abdominal examination revealed a tender, diffuse fullness in the left lumbar region, measuring around 5x6 cm with a localized increase in temperature. The overlying skin was not erythematous but showed areas of altered pigmentation (Figure 1). A soft to firm, non-tender liver was palpable 4 cm below the right costal margin, spanning 14 cm, and the spleen was palpable 3 cm below the left costal margin. No abnormal cardiac or respiratory sounds were detected on auscultation. The possibility of a psoas or a renal abscess, bacterial or tubercular in origin, was considered.

An ultrasonogram (USG) of the left flank showed an ill-defined heterogeneous collection measuring 10 x 6 cm, arising from the posterior interpolar area of the left kidney and extending into perinephric and pararenal spaces, tracking along the lateral renal fascia, with inflammation around the collection and multiple left para-aortic lymph nodes. There was no evidence of nephrolithiasis, hydroureteronephrosis, or any other structural renal tract abnormalities. Blood tests revealed microcytic hypochromic anemia with normal total leucocyte count (76% polymorphs, 12% lymphocytes) and platelet count, raised erythrocyte sedimentation rate, and C-reactive protein. Renal and liver function tests were within normal limits (Table 1). Urine analysis revealed the presence of 20-30 pus cells without any bacteria. The blood and urine cultures were sterile. The patient’s blood tested positive for anti-retroviral antibodies (using 4th generation HIV enzyme-linked immunosorbent assay).

**Table 1:** Laboratory parameters

Parameters	Reference Ranges	Patient Values
Hemoglobin (g/dL)	13-17.0	8.2
Mean corpuscular volume (µm^3^)	76- 100	69.7
Mean corpuscular hemoglobin (pg)	26.0-34.0	23.3
Total leucocyte count (x10^3^/µL)	3.50-10.00	9.85
Platelet count (x10^3^/µL)	150-400	483
Blood urea (mg/dL)	19-45	19
Serum creatinine (mg/dL)	0.7-1.3	0.7
Total serum bilirubin (mg/dL)	0-1	0.2
Total serum protein (g/dL)	6.4-8.3	7.0
Serum albumin (g/dL)	3.5-5.2	2.3
Albumin-globulin ratio	1.2 – 2.0	0.5
Aspartate transaminase (U/L)	<35	10
Alanine transaminase (U/L)	<45	7
Alkaline phosphatase (U/L)	42-128	94
C-reactive protein (mg/L)	<6	104
Erythrocyte sedimentation rate (mm/hr)	<10	53

**Figure 2:**
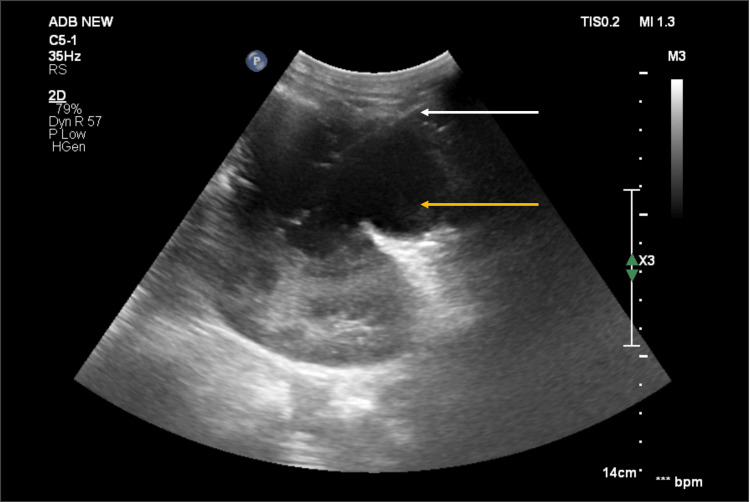
Ultrasonographic image of the renal abscess. The yellow arrow indicates the renal and perirenal collection measuring 10.6 x 6.3 cm. The white arrow points to the pigtail catheter in the abscess cavity.

He was initiated on empirical intravenous antibiotics, ceftriaxone and cloxacillin, to cover for staphylococcal and gram-negative infections. Subsequently, a USG-guided percutaneous aspiration of the abscess drained 60 ml of pus, which on culture grew MRSA. Antibiotics were changed to vancomycin based on the sensitivity pattern. The Mantoux test, pus culture for acid-fast bacilli, and sputum cartridge-based nucleic acid amplification test (CBNAAT) for tuberculosis all yielded negative results. However, seven days after the initial aspiration, the patient experienced worsened left flank pain and swelling. A repeat USG showed a collection of 10.6 x 6.3 cm. Given the significant recollection despite appropriate antibiotics, and considering the patient’s immunocompromised status, a percutaneous indwelling catheter was inserted under USG guidance to ensure continuous drainage (Figure 2).

**Figure 3:**
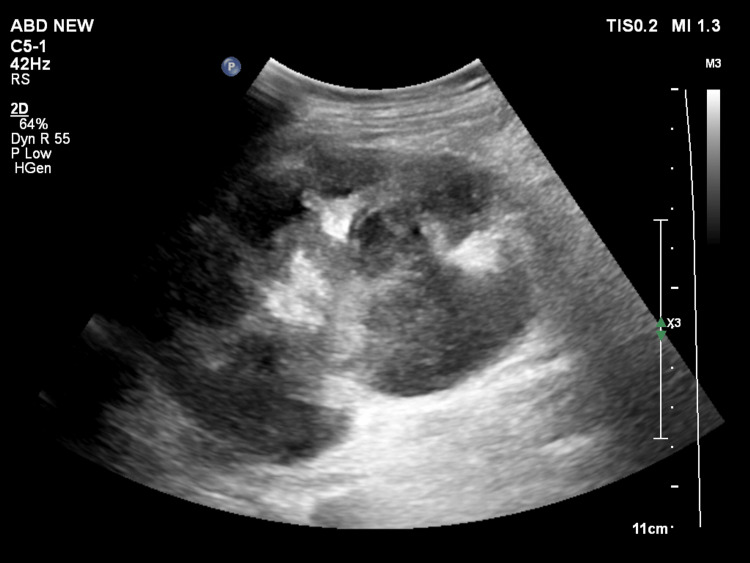
Repeat ultrasonography showing the reniform shape of the kidney after resolution of the abscess cavity with few pockets of intrarenal hypoechoic collection.

In the first 24 hours, approximately 200 ml of pus drained, followed by an additional 220 ml over the next seven days. The pig-tail catheter was eventually removed after eight days when the abscess size had reduced to less than 1.5 cm on USG and the drainage volume had significantly decreased. Transthoracic echocardiogram did not show any evidence of infective endocarditis. The patient’s CD4+ T-cell counts were 226/µL (reference range 381-1565/µL). Highly active antiretroviral therapy was initiated on the 10th day of admission, after ruling out active opportunistic infections. He remained afebrile throughout his hospital stay. The patient received two weeks of in-hospital intravenous vancomycin followed by four weeks of oral linezolid on an outpatient basis. Throughout the treatment, his renal function remained normal. Repeat imaging at six weeks of antibiotic initiation showed resolution of the abscess (Figure 3). While urodynamic studies and functional scans were considered to determine potential underlying structural abnormalities, scarring, and residual renal function, the patient was unfortunately lost to follow-up after three months. 

## Discussion

Renal abscess in children, though rare, is potentially life-threatening. It is characterized by purulent collection within Gerota's fascia or the kidney, often stemming from ascending urinary tract infection (UTI, usually enteric gram-negative bacilli, especially *Escherichia coli*), hematogenous dissemination (usually *S. aureus* from a skin source), or occasionally via direct extension from nearby infected areas. Congenital or acquired renal tract anomalies, including obstruction, stones, vesicoureteral reflux, neurogenic bladder, and instrumentation, may predispose to ascending UTIs. Symptoms are often nonspecific, including fever, abdominal or flank pain, and urinary symptoms [1,2,5,6]. Our patient probably developed a renal abscess through hematogenous spread, as there were no structural predisposing factors on USG.

Given the rarity of renal abscess and the family history, a possible immunocompromised state was considered, which was confirmed upon HIV testing. Vertical transmission from mother to child remains the primary route of pediatric HIV infection, with most untreated cases becoming symptomatic and often resulting in death before five years of age [7,8]. A small minority, however, are asymptomatic, therapy-naive, long-term non-progressors [9]. There are few published reports of adolescent-onset vertically transmitted HIV infection [10-12]. While perinatally acquired HIV cannot be ruled out in our patient, horizontal transmission through minor skin abrasions during caregiving for his affected mother without adequate precautions is a likely possibility in the current case.

Children living with HIV infection frequently experience severe opportunistic infections due to impaired phagocytic cell function and humoral immune abnormalities. Renal abscesses, while uncommon in patients with retroviral infections, can occur, particularly in cases involving* S. aureus*. Community-acquired MRSA has emerged as an important opportunistic pathogen in HIV-infected individuals, with a higher incidence of *S. aureus* bacteremia, especially in those with CD4+ T-cell counts below 200 cells/mm³. Managing staphylococcal infections in these patients often requires aggressive approaches due to the poor treatment response and challenges in eliminating the bacteria, leading to chronic low-grade or recurrent, severe, metastatic infections [3,4]. Effective management involves prompt identification, source removal, appropriate antibiotic therapy, and ruling out potential metastases.

Upon reviewing the retrospective case series of pediatric renal abscesses of the past 20 years, ascending UTIs were identified as the most common etiology; MRSA was found to be a rare causative organism. Though they reported predisposing urogenital abnormalities in several cases, none reported underlying immunodeficiency [2]. The majority of abscesses smaller than 3 cm were successfully managed with antibiotics alone; larger abscesses often necessitated percutaneous or open surgical drainage. Two case reports highlighted staphylococcal renal abscesses in patients with HIV infection. In one instance, a child with HIV infection and chronic *S. aureus* infections developed a renal abscess, which was managed with drainage and six weeks of intravenous antibiotics [13]. Despite having a good CD4 count and being on highly active ART (HAART), the child experienced recurrence after three months, necessitating nephrectomy. Histopathological examination suggested chronic staphylococcal infection progressing to xanthogranulomatous pyelonephritis. Another case involved renal abscess with disseminated metastatic infection, bacteremia, and septic pulmonary emboli, in a patient with a low CD4 count (80 cells/mm^3^), requiring nephrectomy [14]. In our patient, despite immunosuppression, there was no disseminated infection, likely due to a favorable CD4 count. Initial USG-guided percutaneous aspiration was done due to the large size of the collection and to obtain specimens for culture to guide antibiotic therapy. Serial USG examination was utilized for monitoring progress and response to treatment. Subsequent percutaneous catheter insertion was required due to a poor initial response to antibiotics, significant recollection, and an immunocompromised state [1,5,6]. Prompt percutaneous drainage and targeted antibiotic therapy guided by clinical and radiological response averted the need for nephrectomy, resulting in a favorable outcome.

This case emphasizes the importance of considering underlying immunodeficiency in pediatric patients with unusual infections such as renal abscesses. It highlights the necessity for thorough investigation, a multidisciplinary approach, and prompt, tailored treatment. The successful management of this case, including percutaneous drainage and targeted antibiotic therapy, combined with the initiation of HAART, led to a favorable outcome.

## Conclusions

Renal abscesses in children can be life-threatening and often result from UTIs or hematogenous spread. This patient's abscess likely resulted from hematogenous spread due to immunocompromised status. Effective management, including prompt percutaneous drainage and targeted antibiotics, combined with ART, led to abscess resolution and a favorable outcome. This case underscores the importance of considering immunodeficiency in pediatric patients with unusual infections like renal abscesses and highlights the necessity of a multidisciplinary approach and aggressive management for complex infections in immunocompromised children.
